# Magnetic and Microwave Properties of Nanocomposites Containing Iron Particles Encapsulated in Carbon

**DOI:** 10.3390/ma15155124

**Published:** 2022-07-23

**Authors:** Anatoly B. Rinkevich, Dmitry V. Perov, Elena A. Tolmacheva, Evgeny A. Kuznetsov, Olga V. Nemytova, Mikhail A. Uimin

**Affiliations:** M.N. Miheev Institute of Metal Physics UB RAS, Sofia Kovalevskaya St., 18, 620108 Ekaterinburg, Russia; tolmacheva_ea@imp.uran.ru (E.A.T.); kuzeag@mail.ru (E.A.K.); mif--83@mail.ru (O.V.N.); uimin@imp.uran.ru (M.A.U.)

**Keywords:** microwave properties, nanocomposites, transmission and reflection coefficients, ferromagnetic resonance and antiresonance

## Abstract

The magnetic and microwave properties of nanocomposites containing iron particles encapsulated in a carbon shell (Fe@C), as well as carbon nanotubes (CNT), have been experimentally studied. The examination of magnetic properties of composites shows that the materials under study contain a ferromagnetic component. The availability of ferromagnetic ordering for the dielectric matrix-based nanocomposite sample with Fe@C particles has been confirmed by the measurement results of the transmission and the reflection coefficients of the microwaves, since the ferromagnetic resonance has been observed. Furthermore, in the fields less than the field of ferromagnetic resonance, there are the signs of the presence of ferromagnetic antiresonance. The ferromagnetic resonance leads to minima in the transmission and reflection coefficients, whereas the antiresonance, conversely, leads to maxima in the reflection coefficient. The measurement results have been compared with the theoretical calculations of the field dependence of microwave transmission and reflection coefficients.

## 1. Introduction

Carbon-based nanomaterials are one of the crucial materials for most branches of modern technology. A number of works are devoted to composites containing graphene and nanotubes, as well as ferromagnetic particles. In these works, however, magnetic interactions and their effect on microwave phenomena were not studied in detail, and most the attention was paid to the optimization of radio-absorbing properties. A review of microwave absorption in the composites filled with carbonaceous particles was presented in [1].

Particular absorption properties of such composites can be realized by varying the geometry, composition, morphology, and volume fraction of particles. Composites filled with carbonaceous particles are of fundamental interest and relate to a wide range of potential applications, including radar absorption, electromagnetic compatibility for electronic devices, and electromagnetic protection. Production methods of metal particles encapsulated in carbon were described in [2,3]. Such particles are conventionally denoted as Me@C, where Me is a metal. Fe@C and Ni@C particles were prepared by the evaporation of overheated liquid drop of Me in inert gas flow-containing hydrocarbon. Resulting carbon-coated nickel and iron nanoparticles contain metal cores of about 5 nm in size, which are wrapped by a few layers of graphene-like carbon. The particles of Fe@C synthesized by the gas-phase method described above can be used as the magnetic labels number, which can be counted with the aid of the NMR-based method [4]. Carbon coating makes it possible to graft antibodies specific for a particular antigen on it.

The magnetic properties of Fe and Fe_3_C ferromagnetic particles encapsulated in carbon nanotubes were studied in detail in [5,6,7]. The magnetization curves, magnetic anisotropy and the properties of the chains of interacting magnetic particles were investigated. Magneto-optical properties of ferromagnetic metal–carbon nanocomposites belonging to a new class of composites with partial mixing and the formation of metastable phases among components were explored [8]. In the (Co_x_C_1−x_) system, transition from amorphous CoC granules to crystalline Co granules in the carbon matrix occurs while changing the concentration of ferromagnetic granules. The difference of behavior of the magneto-optical properties of (Co_x_C_1−x_) and [(Co_0.4_Fe_0.4_B_0.2_)_x_C_1−x_] systems was explained based on the composite formation model.

An interaction of microwaves with the dielectric spherical particles covered by the conductive shell was modelled [9]. The dependences of the effective permittivity and permeability of powders consisting of core-shell metalized dielectric particles on the frequency, radius, and permittivity of core, as well as thickness and the conductivity of metallic shell, were obtained. For completely conductive particles and dielectric particles having the conductive shell, a contribution of electric and magnetic losses was estimated and compared. The main mechanism of microwave heating of such particles was found to be the dynamic magnetic loss. As electromagnetic wave absorbers, carbon-matrix materials with embedded magnetic nanoparticles can meet the requirements both in terms of magnetic and dielectric [10,11].

The electrodynamics of systems containing carbon nanotubes (CNT) are the subject of a whole series of explorations. In the work [12], the systems of randomly oriented multi-walled nanotubes placed in a rectangular waveguide were experimentally investigated in the frequency range from 8 to 50 GHz. The frequency dispersion of the permittivity was studied. In the nanocomposites consisting of graphene and magnetic particles, an increased absorption of electromagnetic waves is caused both by the electrical losses due to the high conductivity of graphene and by the magnetic losses due to the ferromagnetic particles. Particles of Fe_3_O_4_ [13,14], FeCo alloy, or permalloy [15] are often chosen as magnetic particles. Notice that an interaction between particles increases losses.

Microwave absorption in the arrays of carbon nanotubes functionalized by magnetic particles was described in [16,17]. The best radar absorbing properties were found in the frequency range from 11 to 18 GHz. The combination of high optical transparency and effective absorption of microwaves was achieved for the film systems consisting of the single-walled CNT in the wide enough range of radio and microwave frequencies from 10 MHz to 30 GHz [18]. The correlation of magnetic and radio-absorbing properties was noticed.

In this work, the magnetic properties and absorption of microwaves in nanocomposites containing Fe@C particles and CNT are investigated. Carbon nanotubes raise the microwave conductivity, which enhances microwave losses. In contrast to previous works, the microwave absorption is investigated here as a function of applied magnetic field. To meet the ferromagnetic resonance condition in sufficiently strong magnetic fields, the microwave frequencies from 26 to 38 GHz are chosen to carry out the examinations. The comparison of magnetic and microwave properties of the nanocomposites are carried out. The field dependences of the microwave transmission and reflection coefficients are compared with the theoretical calculations.

## 2. Samples and Methods

Synthesis of Fe@C nanoparticles was described in [2,3]. Briefly, the particles were obtained by the evaporation of overheated (up to 2000 °C) liquid drop of iron in butane-containing argon flow. An iron or nickel seed levitates in high-frequency electromagnetic fields created by an inductor with two sections of turns. At the same time, the drop is heated by currents induced with a coil. The drop begins to evaporate at the temperature reaching about 1800 °C. To replenish the drop, a supply of wire is carried out with the certain speed, which ensures a constancy of temperature. Metal vapors are entrained to a cold zone by an argon stream and condense directly in a carrier gas. When adding hydrocarbons to argon (butane, in our case), the carbon coating forms on the surface of particles.

**Figure 1:**
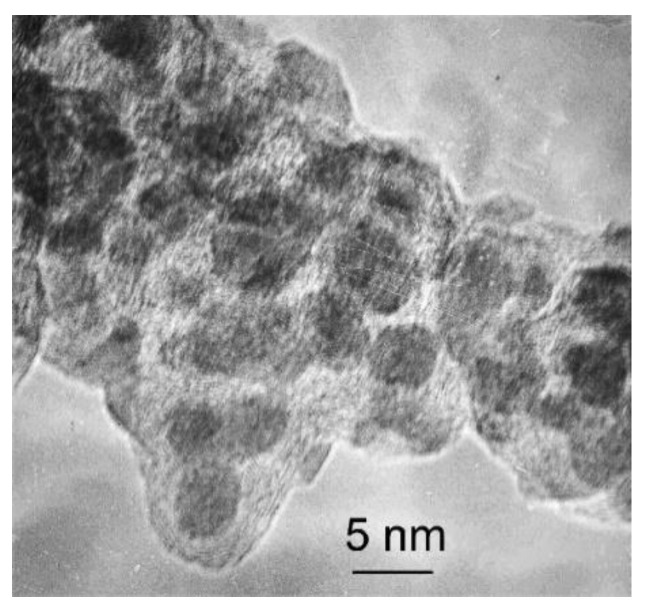
TEM image of Fe@C nanoparticles.

The structure of Fe@C particles was studied using the Philips CM-30 electron microscope. Figure 1 shows that Fe@C particles consist of a core, which is usually darker and a shell. The core size is from 5 to 10 nm, the shell thickness being from 1 to 2 nm. However, the fraction of larger particles up to 17 nm was revealed as well. The carbon shell is not uniform; there are areas with the different thickness from 1–2 to 7 layers.

**Figure 2:**
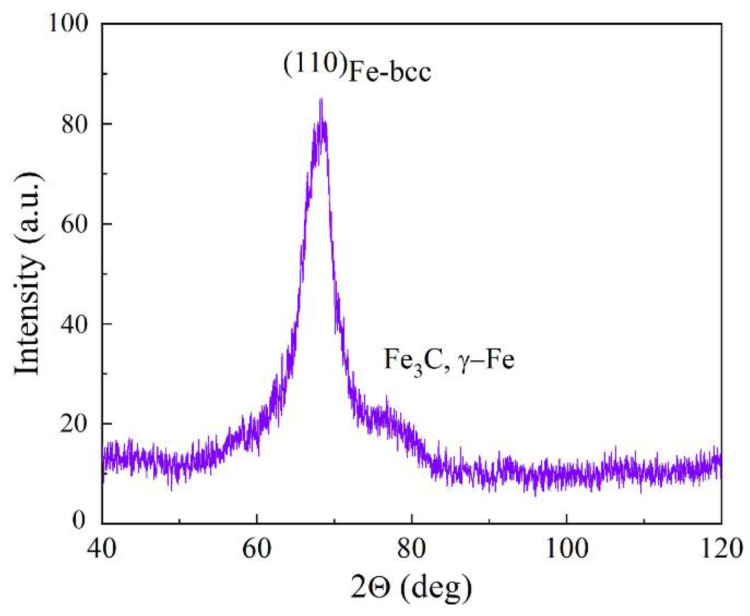
Result of XRD study of Fe@C nanoparticles, obtained with the CrK-*α* radiation.

The coherent-scattering region size was determined from X-ray diffraction (XRD) study. To carry out the investigation, the CrK-α radiation was used. In this radiation the interplanar space of 0.203 nm of (110) bcc-Fe system of planes corresponds to the diffraction angle of 2θ = 68.6°. The result of XRD study is presented in Figure 2. The data obtained confirm the presence of bcc phase and small amount of Fe_3_C carbide. The γ-Fe phase is not clearly visible at the diffraction pattern, because its main peak (111) is imposed on the (110) peak of the α-Fe phase. The other peak of the γ-Fe phase is imposed on the peak coming from the Fe_3_C phase. The same composition was analyzed by a Mössbauer spectroscopy in [19]. The spectra of the Fe_3_C carbide and the doublet corresponding to the Fe(C) phase have been subtracted from the full Mössbauer spectrum. The residual singlet in Figure 1 corresponds to the γ-Fe phase. The composition of the encapsulated powder was found to include γ-Fe (25%), α-Fe (22%), Fe_3_C (24%), as well as an iron-graphite complex (29%). These data do not contradict those obtained by the XRD method.

**Figure 3:**
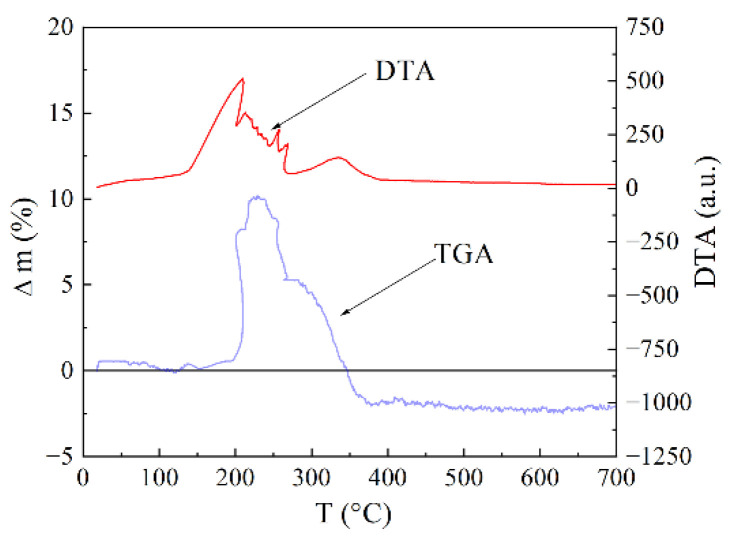
Result of TGA and differential thermal (DTA)** study of Fe@C nanoparticles.

The carbon concentration in the encapsulated particles was determined from thermogravimetric analysis (TGA) results presented in Figure 3. The heating was carried out in the air atmosphere with the rate of 10°/min.

**Figure 4:**
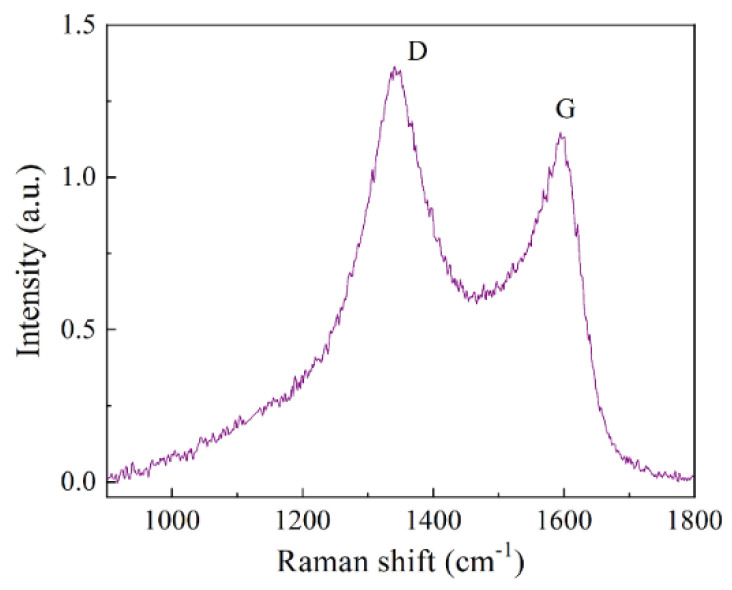
The Raman spectrum for the interval from 950 to 1800 cm^−1^ for Fe@C nanocomposite, measured under excitation at 514 nm.

The mass variation Δm is the joint result of two processes: core oxidation (gain) and carbon combustion (loss). In the temperature range of 150–200 °C, these two processes more or less compensate each other. Then, rapid increase in mass begins. As the shell already passes oxygen well, intensive core oxidation is triggered, which is accompanied by an increase in volume followed by an even greater shell destruction. Further, the shell fragments begin to combust, which leads to a decrease in mass, because the formed CO_2_ exudes together with carbon. Even in the case of ongoing core oxidation, the rate of weight gain is already less than the rate of weight loss caused by the shell combustion. After the combustion of the entire composite, a slight decrease in the sample mass occurs, since the loss of the mass caused by the exuded carbon exceeds the gain caused by the core oxidation. The content of carbon has been obtained by the following method. The mass of the sample M_FeC_ before heating in air. After heating at 1000 °C, whole carbon burns down and Fe_2_O_3_ oxide appears. Then, the sample is weighted again. The mass of iron M_Fe_ is defined according to the chemical formula, namely 69.9% from the mass of Fe_2_O_3_ oxide. If one subtracts the mass M_Fe_ from the primary M_FeC_ mass, then they obtain the carbon mass contained in the FeC sample. As a result, the carbon concentration is found to be 32 wt.%. Raman spectrum in the region of 1000–2000 cm^−1^ contains two lines-D and G near 1340 and 1590 cm^−1^; see Figure 4. In order to estimate the parameters of the carbon envelope, the Raman spectra have been measured for the Fe@C nanocomposite. These spectra have been compared to the spectra of the different carbon materials from graphite to diamond-like carbon films with high content of the tetrahedral bonds [20]. In addition to the main lines near 1340 and 1590 cm^−1^, there are the second rank spectra with peculiarities at ~2600 and 2900 cm^−1^, similar to the glass carbon spectrum. This resemblance points to the fact that the graphene planes are curved and allow the capability to estimate the sizes of the graphene fragments, covering Fe particles. A model of coating structure as the set of curved carbon sheets was proposed based on the analysis of electron microscopy images and Raman spectra [20].

To obtain the mixture of Fe@C and CNT, 31 mg of Fe@C and 20 mg of CNT were taken. In this case, the volume ratio was approximately 2:1. Since both powders have a high degree of aggregation, their mixing was carried out in an ultrasonic bath with alcohol rather than manually. After drying, the mass of the powder was 44 mg. For the microwave measurements, the nanocomposite samples were prepared from the Fe@C powders, as well as from the mixture of Fe@C powder and CNT, blended with epoxy resin. The weight portion of powder was 20%, the volume portion being 7%. After solidification during about a few hours, two samples have the dimensions of 7.2 mm × 3.6 mm × 2 mm with Fe@C and 7.2 mm × 3.6 mm × 10 mm with Fe@C, and the CNT were cut.

**Figure 5:**
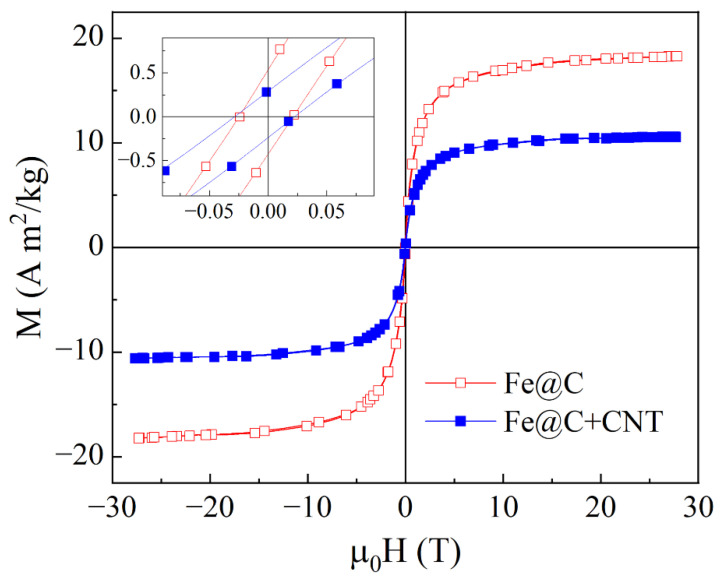
Magnetization curves for the composites containing Fe@C particles and Fe@C + CNT, measured at the room temperature. Inset shows the parts of the hysteresis loops. Here and further $\mu_{0}$ = 4π × 10^−7^ H/m is the magnetic constant.

To define the magnetic state of Fe@C particles, the magnetic measurements were performed in the range of magnetic fields up to 28 kOe at room temperature. The results for nanocomposites with Fe@C particles and Fe@C + CNT are shown in Figure 5. 

For the sample with Fe@C particles, the magnetization *M* sharply increases when applying the magnetic field *H*. The saturation occurs in the fields of about 0.54 T. For the sample with Fe@C + CNT mixture, the magnetization value decreases in proportion to the fraction of Fe@C particles. Notice that despite the small size of particle core, this material does not demonstrate superparamagnetic properties. The inset in Figure 5 shows that there are narrow hysteresis loops, so the nanocomposites behave like a ferromagnet. The coercivity for both samples are about 3 mT. The question is why the system containing such small particles behaves as a ferromagnetic one rather than superparamagnetic. The most prominent reasons of this phenomenon are following: the exchange interaction between the contact particles, the presence of large ferromagnetic particles and the increased anisotropy of metal-carbon interface, the particle core inhomogeneously saturated with carbon. Within the framework of this paper, we did not make the conclusion about ferromagnetism nature in our systems. To understand the results of microwave measurements, the fact of the presence of ferromagnetism by themselves is important.

## 3. Microwave Measurements and Results

**Figure 6:**
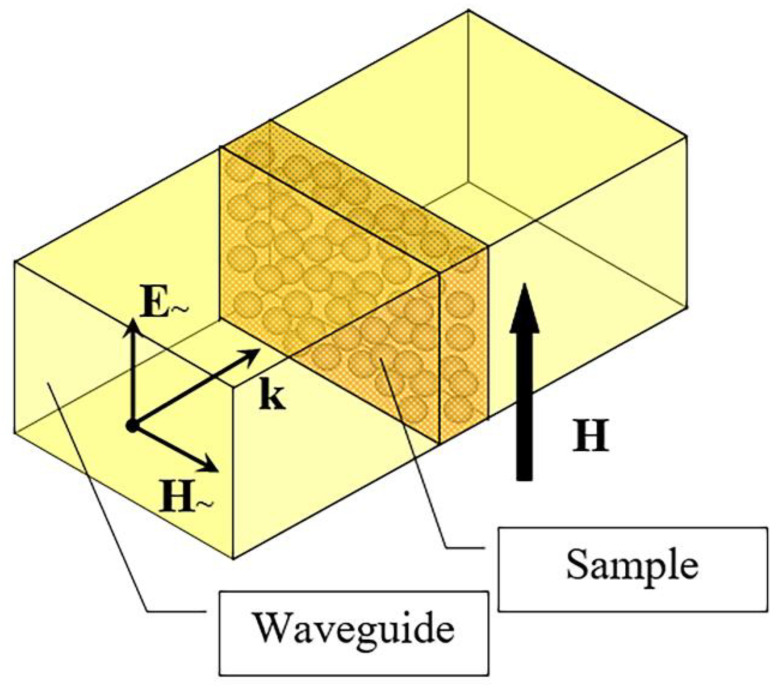
Scheme of microwave measurements.

The microwave measurements were performed in the frequency range from 26 to 38 GHz, using a panoramic scalar network analyzer. One of the purposes of the microwave measurements was to determine the permittivity *ε* and conductivity *σ* of the nanocomposites, $\varepsilon =\varepsilon^{\prime }-i\frac{\sigma }{\omega \varepsilon_{0}}$, where *ω* = 2π*f* is the angular frequency, the electric constant $\varepsilon_{0}\approx$ 8.854 × 10^−12^ F/m. To perform the measurements, the nanocomposite sample was placed into the cross section of standard rectangular waveguide with the dimensions of 7.2 mm × 3.4 mm and completely overlaps it. Figure 6 shows the microwave experiment scheme. To prevent the microwave leakage, the side surfaces of the sample were smeared with conductive glue. All microwave experiments were carried out at room temperature.

The modules of transmission *T* and reflection *R* coefficient were measured. To determine the numerical values of permittivity and conductivity, the technique described in [21] and briefly described below was used.

The real parts of the effective permittivity *ε* and microwave conductivity *σ* are estimated using two measured characteristics—the modules of the transmission and reflection coefficients. The difference between the theoretically calculated and measured values of transmission coefficient modules is computed and minimized by the least square method. The values of the effective permittivity *ε*′ and conductivity *σ* resulted in a minimization procedure that can be considered as the estimation results. This method was successfully applied to estimate the permittivity of ceramic and the nanocomposite titanates of transition metals [22] and analyze the millimeter waveband dielectric properties of the nanocomposite materials based on the opal matrices with spinel particles [23].

**Figure 7:**
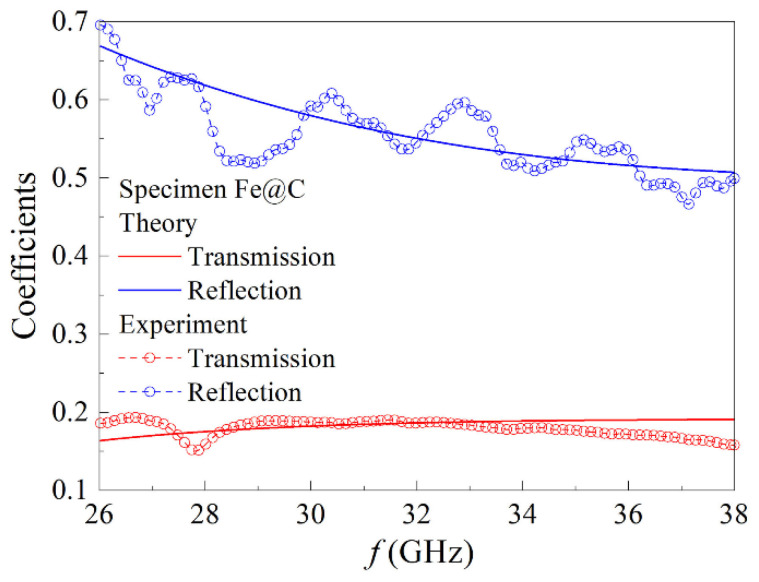
Frequency dependences of the transmission and reflection coefficients in the millimeter waveband and their approximations for the nanocomposite sample of 2 mm in thick with Fe@C particles.

The experimentally measured frequency dependences of the transmission and reflection coefficients for the nanocomposite sample of 2 mm in thick with Fe@C particles are shown in Figure 7. The dependences calculated by the formulas given in [21] for optimally chosen values of *ε*′ and *σ* are represented in this figure by thicker lines. Because of the slight unavoidable mismatch of waveguide tract, there are the coefficient oscillations in the experimental data. In a whole, the calculated and measured dependences are close to each other. According to the estimation carried out, the effective permittivity equals to 4.8 ± 0.2, and the value of effective conductivity equals to 8.5 ± 0.9 S/m. These values are typical for lossy dielectric and this is not surprising, since the nanocomposite is based on the dielectric matrix, and the concentration of the conductive particles is below the percolation threshold.

**Figure 8:**
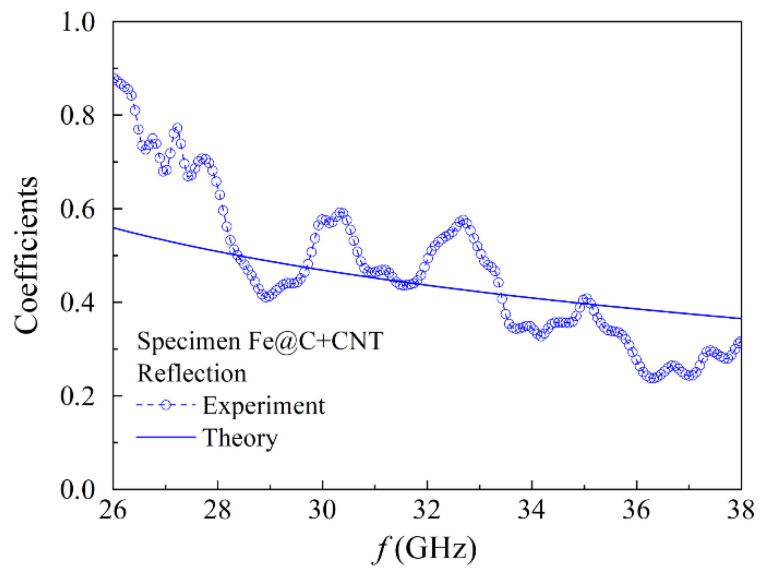
Frequency dependence of the reflection coefficient in the millimeter waveband for the nanocomposite sample of 10 mm in thick with Fe@C particles and CNT.

For the nanocomposite sample of 10 mm in thick with Fe@C particles and CNT, the transmission coefficient is small. Figure 8 represents the frequency dependence of the reflection coefficient. As a whole, the reflection coefficient decreases when increasing the frequency. Based on the earlier mentioned algorithm with the aid of the frequency dependence of the reflection coefficient, the effective permittivity and microwave conductivity were calculated, the values being *ε*′ = 2.7 and *σ* = 14.3 ± 1.4 S/m, correspondingly. This dependence has the oscillatory manner caused by the slight mismatch of the microwave tract. 

Since the nanocomposites contain the ferromagnetic particles of iron, the ferromagnetic resonance (FMR) and possibly antiresonance (FMAR) phenomena are expected to occur. The FMR phenomenon consists in the absorption of microwaves, providing that the wave frequency and external DC magnetic field satisfy the relation [24]

(1) $$\omega =\gamma \mu_{0}\sqrt{H{\left(H+M_{s}\right)}^{}}\;\mathrm{for}\;H>H_{s}$$

which is valid for the fields *H* exceeding the field of magnetic saturation *H_s_*. In Formula (1) *γ* is the gyromagnetic ratio, $M_{s}$ is the saturation magnetization. 

Equation (1) is written for a plate-shaped sample and an external magnetic field applied in the direction shown in Figure 6. In the microwave experiments carried out in the external DC magnetic field, the relative variations of the transmission coefficient module $t_{m}=\frac{\left|T\left(H\right)\right|-\left|T\left(0\right)\right|}{\left|T\left(0\right)\right|}$, where |T(H)| is the transmission coefficient module, as well as the relative variations of the reflection coefficient module $r_{m}=\frac{\left|R\left(H\right)\right|-\left|R\left(0\right)\right|}{\left|R\left(0\right)\right|}$ where |R(H)| is the reflection coefficient module, were measured.

**Figure 9:**
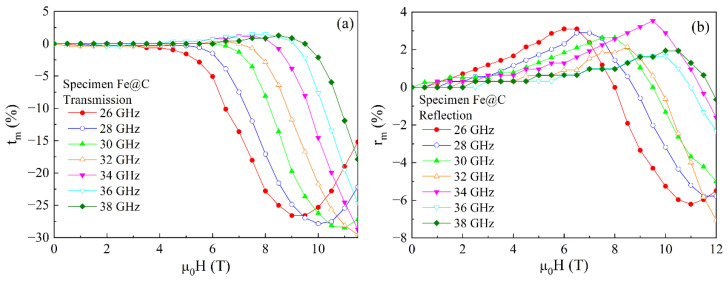
Magnetic field dependences of the transmission (**a**) and reflection (**b**) coefficients measured at several frequencies of the millimeter waveband for the sample of 2 mm thick with Fe@C particles.

The field dependences of the transmission coefficient for the sample of 2 mm in thick with Fe@C particles are presented in Figure 9a. The dependences measured at frequencies of up to 30 GHz have the minima. At the higher frequencies, the minima are not achieved.

Figure 9b shows the similar dependences for the reflection coefficient with the minima caused by the absorption of microwaves upon the fulfillment of ferromagnetic resonance condition; see Formula (1). The size of particle with the carbon shell does not exceed 20 nm, and the skin depth at the frequency of 30 GHz is about 1 μm. Since the particle size is much smaller than the skin depth, the conductivity of Fe@C particles insignificantly influences the FMR. Notice that the FMR line width is large, no less than 0.4 T. In the dependences of the reflection coefficient in fields less than the FMR field, besides the minima, there are the maxima as well. Whereas for the dependences of the transmission coefficient, the maxima appear at the frequencies over 30 GHz. These maxima are caused by the fulfillment of the FMAR condition, upon which the real part of the effective microwave permeability is zero. 

The FMAR phenomenon was initially observed for the ferromagnetic metallic films [24]. The equality of the real part of the permeability to zero leads to the sharp increase in the skin depth. Therefore, the transmission of waves through the film increases with the FMAR conditions. For the nanocomposites based on the dielectric matrices, the FMAR was experimentally found to lead to the increase in the reflection coefficient [25]. Figure 9 shows that the maxima caused by the FMAR are observed both in the reflection coefficient dependences for all frequencies and in the transmission coefficient dependences for the frequencies higher than definite frequency, namely 30 GHz. The FMAR fields are less than the FMR fields. 

**Figure 10:**
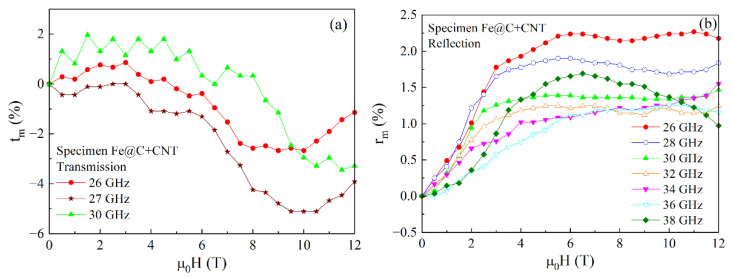
Magnetic field dependences of the transmission (**a**) and reflection (**b**) coefficients, measured at several frequencies of the millimeter waveband for the sample of 10 mm thick with Fe@C particles and CNT.

Figure 10 represents the field dependences of the transmission and reflection coefficient for the nanocomposite with Fe@C particles and CNT. Because of the large absorption at the higher frequencies, the dependences of the transmission coefficient were measured only at the frequencies of 26, 27, and 30 GHz. In the dependences shown in Figure 10a, there are the minima caused by the FMR, which position shifts toward the region of the higher fields when increasing the frequency. In the dependences of the reflection coefficient shown in Figure 10b, there are the maxima that are probably caused by the FMAR phenomenon. The next section of the article presents the results of calculations, as well as the comparative analysis of the calculation and experimental data.

## 4. Discussion

The propagation of electromagnetic waves in a ferromagnetic medium was considered, in particular, in monograph [24], and specificity of a microinhomogeneous media was studied in [26,27,28,29]. In the configuration $H⊥H_{\sim }$ considered in this article, the formula for the wave number of the electromagnetic wave in a magnetized medium is given by:

(2) $$k=k_{0}\sqrt{\varepsilon^{\prime }\;\left(\mu_{xx}-\frac{\mu_{xy}\mu_{yx}}{\mu_{yy}}\right)}=k_{0}\sqrt{\varepsilon^{\prime }\;\mu_{eff}}$$

where $k_{0}=\frac{\omega }{c}$ is the free space wavenumber, $c=\frac{1}{\sqrt{\varepsilon_{0}\mu_{0}}}$, and the effective permeability $\mu_{eff}$ is introduced as follows:

(3a) $$\mu_{eff}=\mu_{xx}-\frac{\mu_{xy}\mu_{yx}}{\mu_{yy}}$$

(3b) $$\langle \mu_{eff}\rangle =\langle \mu_{eff}\left(\Theta \right)\rangle =\langle \mu_{xx}^{m}\left(\Theta \right)-\frac{\mu_{xy}^{m}\left(\Theta \right)\cdot \mu_{yx}^{m}\left(\Theta \right)}{\mu_{yy}^{m}\left(\Theta \right)}\rangle$$

Formula (3a) is written for a homogeneous medium and (3b) for a composite one. Here Θ=(α,β,γ) is the vector of rotation angles of a ferromagnetic particle relative to the *x*, *y*, *z* axes. The angle brackets in (3b) stand for the angle Θ averaging. In the case of a homogeneous medium, the following relations are valid for the classical Polder tensor: $\mu_{xx}=\mu_{yy}=\mu$, $\mu_{xy}=i\mu_{a}$, $\mu_{yx}=-i\mu_{a}$. In this case, Formula (3b) takes the known form [23]:

(3c) $$\mu_{eff}=\mu -\frac{\mu_{a}^{2}}{\mu }$$

We believe that the composite medium in any elementary volume contains the same volume fraction of ellipsoidal ferromagnetic particles $\theta_{v}$, the size of each particle being much less than the electromagnetic wavelength. According to [28], the formula for ${\overset{↔}{\mu }}^{m}$ of the composite medium is given by

(4) $$\langle {\overset{↔}{\mu }}^{m}\rangle =\left(1-\theta_{v}\right)\cdot \overset{↔}{I}+\theta_{v}\cdot \langle \overset{↔}{\mu }\rangle =\langle \left(\begin{array}{ccc} \mu_{xx}^{m}\left(\Theta \right) & \mu_{xy}^{m}\left(\Theta \right) & 0\\ \mu_{yx}^{m}\left(\Theta \right) & \mu_{yy}^{m}\left(\Theta \right) & 0\\ 0 & 0 & 1 \end{array}\right)\rangle$$

In Formula (4), $\overset{↔}{I}$ is the second rank unit tensor. The dependence of the components of the effective magnetic permeability tensor on the external magnetic field, the frequency and fraction of ferromagnetic particles are expressed by Formula (5):

$$\mu_{xx}^{m}\left(\Theta \right)=1+\frac{\theta_{v}\omega_{M}\left(\Theta \right){\left[\omega_{H}+i\omega \alpha -\left({\tilde{N}}_{zz}\left(\Theta \right)-{\tilde{N}}_{yy}\left(\Theta \right)\right)\left(1-\theta_{v}\right)\omega_{M}\left(\Theta \right)\right]}^{}}{\hat{D}\left(\Theta \right)}$$

$$\mu_{xy}^{m}\left(\Theta \right)=\frac{\theta_{v}\omega_{M}\left(\Theta \right){\left[i\omega -{\tilde{N}}_{xy}\left(\Theta \right)\left(1-\theta_{v}\right)\omega_{M}\left(\Theta \right)\right]}^{}}{\hat{D}\left(\Theta \right)}$$

$$\mu_{yx}^{m}\left(\Theta \right)=-\frac{\theta_{v}\omega_{M}\left(\Theta \right){\left[i\omega +{\tilde{N}}_{xy}\left(\Theta \right)\left(1-\theta_{v}\right)\omega_{M}\left(\Theta \right)\right]}^{}}{\hat{D}\left(\Theta \right)}$$

$$\mu_{yy}^{m}\left(\Theta \right)=1+\frac{\theta_{v}\omega_{M}\left(\Theta \right){\left[\omega_{H}+i\omega \alpha -\left({\tilde{N}}_{zz}\left(\Theta \right)-{\tilde{N}}_{xx}\left(\Theta \right)\right)\left(1-\theta_{v}\right)\omega_{M}\left(\Theta \right)\right]}^{}}{\hat{D}\left(\Theta \right)}$$

$$\overset{⌢}{D}\left(\Theta \right)=\left[\omega_{H}+i\omega \alpha -\left({\tilde{N}}_{zz}\left(\Theta \right)-{\tilde{N}}_{xx}\left(\Theta \right)\right)\left(1-\theta_{v}\right)\omega_{M}\left(\Theta \right)\right]\cdot \;\cdot \left[\omega_{H}+i\omega \alpha -\left({\tilde{N}}_{zz}\left(\Theta \right)-{\tilde{N}}_{yy}\left(\Theta \right)\right)\left(1-\theta_{v}\right)\omega_{M}\left(\Theta \right)\right]\cdot \;-{\left({\tilde{N}}_{xy}\left(\Theta \right)\left(1-\theta_{v}\right)\omega_{M}\left(\Theta \right)\right)}^{2}-\omega^{2}$$

(5) $$\overset{↔}{\tilde{N}}\left(\Theta \right)=\left(\begin{array}{ccc} {\tilde{N}}_{xx}\left(\Theta \right) & {\tilde{N}}_{xy}\left(\Theta \right) & {\tilde{N}}_{xz}\left(\Theta \right)\\ {\tilde{N}}_{xy}\left(\Theta \right) & {\tilde{N}}_{yy}\left(\Theta \right) & {\tilde{N}}_{yz}\left(\Theta \right)\\ {\tilde{N}}_{xz}\left(\Theta \right) & {\tilde{N}}_{yz}\left(\Theta \right) & {\tilde{N}}_{zz}\left(\Theta \right) \end{array}\right)$$

where $\omega_{H}=\gamma \mu_{0}H_{z}$, $\omega_{M}\left(\Theta \right)=\gamma \mu_{0}M_{z}\left(\Theta \right)$, *α* is the dimensionless damping constant in a magnetic system. Formula (5) take into account the dependence of the components of ellipsoidal particle demagnetizing tensor $\overset{↔}{\tilde{N}}\left(\Theta \right)$ both upon the variation of the orientation of the particle, as well as the value of $M_{z}\left(\Theta \right)$ and, therefore, $\omega_{M}\left(\Theta \right)$. Note that $\mathrm{tr}\overset{↔}{\tilde{N}}\left(\Theta \right)=1$. The parameter $M_{z}\left(\Theta \right)$ denotes the equilibrium value of the z component of the constant magnetization vector corresponding to a particle oriented in accordance with the vector Θ. Obviously, $M_{z}\left(\Theta \right)\leq M_{s}$ for any Θ.

**Figure 11:**
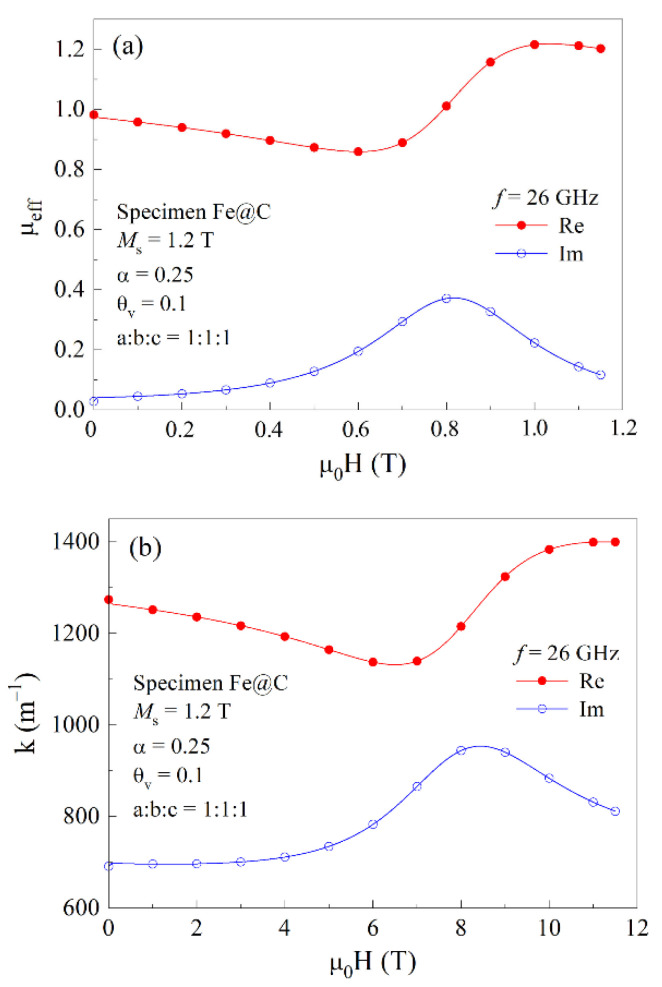
Magnetic field dependences of the real and imaginary parts of the effective permeability (**a**) and wave number (**b**) for the composite containing 7% of spherical ferromagnetic particles at the frequency of 26 GHz.

The calculation details of the components of the effective permeability tensor for a composite are given in [28]. We performed numerical calculations of the magnetic field dependence of the effective permeability for a composite containing spherical particles with a radius of 25 nm, with the volume fraction of ferromagnetic particles being $\theta_{v}$ = 0.07. The calculated material constants of the composite coincide with those measured for the sample of 2 mm in thick: *ε*′ = 4.75, *σ* = 8.54 S/m, *α* = 0.25. The X-ray and Mössbauer data show that most of iron is not chemically bound to other elements, the Fe_3_C fraction being less than a quarter. Therefore, for the further calculations, the magnetization of *α*-Fe $M_{s}$ = 1.2 T. Figure 11a shows the calculation results for the frequency of 26 GHz.

The maximum in the dependence of the imaginary part of the permeability in Figure 11a is caused by the absorption of microwaves while obeying the FMR condition (1). The field dependence of the real and imaginary parts of the wave number is shown in Figure 11b. As is seen, the real part of the wave number is greater than imaginary, $k^{\prime }>k^{\prime \prime }$. This ratio indicates that this composite is the lossy dielectric.

The transmission T and reflection R coefficients can be given by formulas [28,30]:

(6) $$T=\frac{2Z_{1}Z_{2}}{2Z_{1}Z_{2}\cos\;\left(k_{2}d_{2}\right)+i\;\left(Z_{1}^{2}+Z_{2}^{2}\right)\;\sin{\left(k_{2}d_{2}\right)}^{}{}^{}}$$

(7) $$R=\frac{i\;{\left(Z_{2}^{2}-Z_{1}^{2}\right)}_{}^{}\sin\left(k_{2}d_{2}\right)}{2Z_{1}Z_{2}\cos\;\left(k_{2}d_{2}\right)+i\;\left(Z_{1}^{2}+Z_{2}^{2}\right)\;\sin{\left(k_{2}d_{2}\right)}^{}{}^{}}$$

Here, $Z_{1}=\sqrt{\frac{\mu_{0}}{\varepsilon_{0}}}$ is the impedance of free space, $Z_{2}=\sqrt{\frac{\mu_{0}\langle \mu_{eff}\rangle }{\varepsilon_{0}\varepsilon }}$ is the impedance of the composite. The power transmission $T_{P}$ and reflection $R_{P}$ coefficients show the portions of microwave power in the wave passed the plate and reflected from it:

(8) $$T_{P}=T\cdot T^{*},\;R_{P}=R\cdot R^{*}$$

Dissipation of microwave power *D* can be calculated from these coefficients as follows:

(9) $$D=1-T_{P}-R_{P}$$

The dissipation indicates the fraction of power lost within the plate because of the absorption, as well as the scattering on inner inhomogeneities. Of course, the dissipation can depend on the magnetic field.

**Figure 12:**
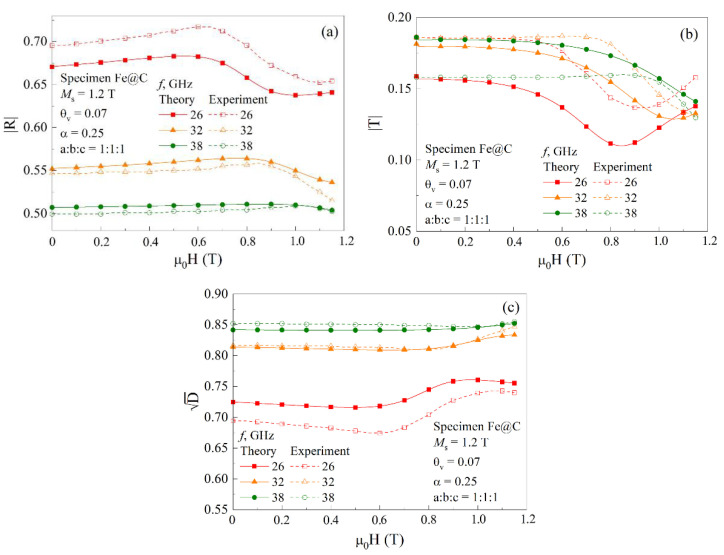
Comparison of the calculated and measured dependences of the reflection (**a**), transmission (**b**) and dissipation (**c**) coefficients for the composite containing 7% spherical ferromagnetic particles at the frequencies of 26, 32, and 38 GHz.

Figure 12 shows the experimental and calculated field dependences of the reflection (a), transmission (b) and power dissipation (c) coefficients for the composite containing 7% of spherical ferromagnetic particles at the frequencies *f* = 26, 32, and 38 GHz. One can conclude that within the framework of the approximations made, the calculation satisfactorily describes both the magnitude of the coefficients and their field dependence. Both in the calculated and experimentally obtained dependences of the reflection and transmission coefficients, there are minima caused by the microwave power absorption while fulfilling the FMR condition. The calculated field of minimum approximately equals to the experimentally determined one for each frequency. In the dependences of the reflection coefficient, the maxima in the fields, lower than the FMR field, are clearly visible. These maxima are caused by the realization of the FMAR in the nanocomposite sample. The field dependence of the dissipation is presented in Figure 12c. The fields of the dissipation maximums correspond to the FMR condition. Figure 12c shows that the calculation carried out describes the characteristic features of the experimentally measured dependences. Certain mismatches of *D* values at the frequency of 26 GHz are possibly caused by a weakly field-dependent contribution to the dissipation, because of the wave scattering on inhomogeneities in the composite sample, that does not take into account in the calculation.

## 5. Conclusions

The microwave and magnetic properties of the nanocomposites containing Fe@C particles and CNT mixed with epoxy resin have been investigated. The study of the magnetic properties of the samples under consideration shows the presence of the ferromagnetic ordering that manifests itself by the presence of the hysteresis loop. The measured frequency characteristics of the transmission and reflection coefficients have been used to estimate the real part of the effective permittivity and conductivity of the nanocomposite containing Fe@C particles in the frequency range from 26 to 38 GHz. 

The microwave measurements carried out in the external DC magnetic field confirmed the ferromagnetic ordering of the nanocomposites, since the ferromagnetic resonance has been observed. For both nanocomposites, the signs of presence of the ferromagnetic antiresonance have been observed in fields smaller than the FMR field. The FMAR has been firstly realized for the nanocomposites containing ferromagnetic components and nano-modification of carbon. The detailed comparison of the experimental results with the theory based on the concept that the composite is a medium with the effective parameters has been carried out. The comparison showed that, in spite of the accepted simplifications, the microwave properties of the composite in the external DC magnetic field are described by the theory correctly.

## Data Availability

Not applicable.
